# Differences in the Impact of Obesity and Bariatric Surgery on Patients Hospitalized for Atrial Flutter and Atrial Fibrillation: A Nationwide Analysis, 2016-2020

**DOI:** 10.7759/cureus.62284

**Published:** 2024-06-13

**Authors:** Ricardo Machado Carvalhais, Christian Siochi, Gohar Harutyunyan, Danny Segura Torres, Vahe Shahmoradi, Peter Sobieraj, Aressa Canuto Miller, Stephen Jesmajian

**Affiliations:** 1 Internal Medicine, Montefiore New Rochelle Hospital, Albert Einstein College of Medicine, New Rochelle, USA

**Keywords:** metabolic and bariatric surgery (mbs), national inpatient sample (nis), post-bariatric surgery, endotracheal intubation, cardioversion, inpatient mortality, obesity paradox, obesity, atrial flutter, atrial fibrillation (af)

## Abstract

Background: The "obesity paradox" claims that although obesity is a risk factor for atrial fibrillation, obese patients have lower inpatient mortality when admitted due to atrial fibrillation. This study aims to analyze if the obesity paradox still holds true after weight loss from bariatric surgery.

Methods: This study analyzed discharge data from the National Inpatient Sample, 2016-2020. Patients admitted due to atrial fibrillation or atrial flutter, with or without obesity, and with or without a past medical history of bariatric surgery were identified using ICD-10-CM and ICD-10-PCS codes. The primary outcome was mortality. Secondary outcomes included length of stay, resource utilization, necessity for endotracheal intubation, and necessity for cardioversion. STATA v.13 was used for univariate and multivariate analysis (StataCorp LLC, Texas, USA).

Results: Among 2,292,194 patients who had a primary diagnosis of atrial fibrillation or atrial flutter, 494,830 were obese and 25,940 had bariatric surgery. Mortality was not significantly different in post-bariatric surgery patients when compared to the general population (OR 0.76; 95% [CI 0.482-1.2; p=0.24]). Mortality was significantly lower in obese patients when compared to the general population (OR 0.646; 95% [CI 0.583-0.717; p<0.001]). Therefore, post-bariatric surgery patients had a higher mortality than obese patients when compared to the general population. Obese patients spent more days in the hospital (regression 0.219; 95% [CI 0.19-0.248, p<0.001]), had higher resource utilization (regression 3491.995; 95% [CI 2870.085-4113.905, p<0.001]), more cardioversions (OR 1.434; 95% [CI 1.404-1.465; p<0.001]), and no difference in endotracheal intubation rate (OR 1.02; 95% [CI 0.92-1.127; p=0.724]) when compared to the general population. Post-bariatric patients had no difference in length of stay (regression -0.053; 95% [CI -0.137-0.031; p=0.218]) and resource utilization (regression 577.297; 95% [CI -1069.801-2224.396; p=0.492]), fewer endotracheal intubations (OR 0.583; 95% [CI 0.343-0.99; p=0.046]), and more cardioversions (OR 1.223; 95% [CI 1.134-1.32; p<0.001]) when compared to the general population.

Conclusion: Compared to the general population, post-bariatric patients had higher inpatient mortality than obese patients when admitted due to atrial fibrillation or atrial flutter. This research reinforces the presence of the obesity paradox following bariatric surgery with respect to mortality.

## Introduction

Atrial fibrillation and obesity

Atrial fibrillation (AF) and obesity are increasingly prevalent. As of 2016, there are approximately 5.2 million cases of AF in the United States, and this number is projected to rise to 12.1 million by 2030 [1]. The World Health Organization (WHO) reported in 2016 that the prevalence of obesity has nearly tripled since 1980 [2]. 

The increasing prevalence of obesity is a significant contributing factor to the global burden of chronic non-communicable diseases. In epidemiological studies, obesity is often defined using body mass index (BMI) as a standardized measure that allows for comparisons across different populations and research studies. According to the World Health Organization's criteria, individuals with a BMI of 25 kg/m2 or higher are considered overweight, while those with a BMI of 30 kg/m2 or higher are classified as obese [2]. Obesity has been linked to various health problems such as cardiovascular diseases, diabetes, premature death, and certain types of cancer [3, 4]. 

Given the increasing prevalence of AF and its associated complications, there is a pressing need for effective management and prevention strategies. AF is a condition characterized by irregular and rapid heartbeats which can lead to stroke, heart failure, and death. It is the most common cardiac arrhythmia in adults, with approximately 600,000 visits to the emergency department each year in the United States alone [5]. Of those who seek emergency care for AF, over 60% require admission to inpatient units [5]. The condition contributes to a substantial number of hospitalizations, as many individuals with AF require inpatient care for proper management and treatment. 

Various factors related to obesity contribute to the widespread occurrence of atrial fibrillation. Chronic obesity is associated with a range of complications such as obstructive sleep apnea, metabolic syndrome, hypertension, diabetes, and coronary heart disease [6]. These complications play a crucial role in the remodeling of the atria and contribute to the initiation and perpetuation of AF [7]. The cumulative impact of obesity-related complications increases the workload on the heart, resulting in higher oxygen consumption by the myocardium [8]. This elevated myocardial metabolism can lead to the production of reactive oxygen species (ROS) such as superoxide, hydrogen peroxide, and the hydroxyl radical during mitochondrial respiration. When the production of ROS exceeds the antioxidant capacity of the cell, oxidative stress occurs. Elevated levels of oxidative stress can subsequently lead to impaired cardiac contractility, ultimately resulting in arrhythmias [8]. Genetics may also play a role. A study utilizing Mendelian randomization to investigate the causal relationship between obesity and AF found that a genetic risk score comprising 39 body mass index polymorphisms was significantly associated with AF [9].

Obesity paradox

Numerous studies have demonstrated an “obesity paradox” where overweight and obese patients with AF have a better prognosis than leaner patients with the same degree of severity of AF [10-12]. In the Gulf-SAFE registry study conducted by Li et al., a multivariate analysis was performed to determine the potential association between obesity and the risks of stroke, bleeding, heart failure admission, and a composite outcome in patients with AF [13]. Their study included a total of 1,804 AF patients with body mass index considered in the final analysis. The average age of the participants in their analysis was 56.2 ± 16.1 years, and 47.0% of them were female. Among their participants, 559 individuals (31.0%) were classified as obese with a BMI over 30 kg/m2. Their patients were followed for a period of 12 months. Their findings revealed an intriguing correlation between higher BMI and reduced risks of the aforementioned conditions, as well as all-cause mortality. Surprisingly, obesity and higher BMI were associated with a more favorable prognosis; their results highlight the existence of an obesity paradox. 

Another study conducted by Zhu et al. suggested that in patients with AF, having an underweight BMI is associated with poorer outcomes while being overweight or obese does not elevate the risk, thus supporting the presence of an obesity paradox [14]. Their meta-analysis approach investigated the correlation between BMI and clinical outcomes in patients with AF. Their research involved nonvalvular AF patients from randomized controlled trials or observational studies that classified BMI based on WHO standards. They examined a total of 49,364 participants from nine studies, with outcomes including stroke or systemic embolism (SSE), all-cause death, and cardiovascular death. Their findings revealed that being underweight was linked to higher risks, whereas being overweight or obese did not show an increased risk of SSE, cardiovascular death, or all-cause death in AF patients. 

Bariatric surgery

Surgical techniques used to treat obesity are referred to as metabolic or "bariatric" procedures. Metabolic and bariatric surgery (MBS) are some of the fastest-growing operative procedures performed worldwide [15]. MBS affects weight loss through three fundamental mechanisms: malabsorption, restriction, and neurohormonal response that regulates hunger and energy balance [16]. 

Study aim

This study focuses on hospitalized patients who were admitted due to atrial flutter or AF to analyze the impact of obesity and bariatric surgery. This study aims to investigate if the obesity paradox still holds true after bariatric surgery. This article was previously presented as a poster contribution at the 2024 American College of Cardiology Scientific Session on April 6, 2024. 

## Materials and methods

Study design

This was a retrospective cohort study using discharge data from the National Inpatient Sample (NIS), Healthcare Cost and Utilization Project (HCUP), Agency for Healthcare Research and Quality from 2016 to 2020. 

Study inclusion criteria

Patients with a non-elective admission primary diagnosis of AF or atrial flutter, aged > 18 years, with or without obesity, and with or without a history of bariatric surgery were identified using International Classification of Disease, Tenth Edition, Clinical Modification (ICD-10-CM) and Procedure Coding System (ICD-10-PCS) codes. 

Ethical considerations 

The data from the NIS-HCUP is publicly available and de-identified, hence exempt from institutional review board approval. The need for informed consent was waived.

Outcome measures 

The primary outcome of interest is in-hospital mortality, while secondary outcomes include length of stay, resource utilization, the need for endotracheal intubation, and the need for cardioversion. 

Statistical analysis 

This study used a confidence interval (CI) of 95% and a p-value <0.05 as statistically significant in its analysis. Continuous variables were examined through the calculation of means accompanied by standard deviations or medians along with interquartile ranges in the case of normally distributed and skewed data, respectively. Descriptive statistics incorporating frequencies and percentages were employed for the analysis of categorical variables. Patient and hospital-level characteristics and in-hospital outcomes were compared between patients with AF or atrial flutter, with or without obesity who underwent bariatric surgery using the Pearson χ2 test for categorical variables and the independent sample t-test for continuous variables. To calculate unadjusted and adjusted odds ratios for in-hospital clinical outcomes, univariate and multivariate logistic regression were used. To account for potential confounding factors, a multivariate regression model was adjusted for patient and hospital-level baseline characteristics. All analyses were conducted using STATA v.13 TX (StataCorp, LLC, Texas, USA).

## Results

Baseline patient characteristics, including demographics and Charlson comorbidity index, and hospital-level characteristics are shown in Table 1. A total of 2,292,194 patients, aged >18 years with a non-elective admission primary diagnosis of AF or atrial flutter in the United States between 2016 and 2020 were included in the final cohort. In the final cohort, 494,830 were classified as obese and 25,940 underwent bariatric surgery. The mean age of obese and non-obese patients was 64.53 and 72.27, respectively. The mean age of bariatric surgery and no bariatric surgery patients was 63.02 and 70.68, respectively (Table 1). The majority of patients with and without obesity were male, 52.54% and 50.62%, respectively. The majority of patients with bariatric surgery were female (63.87%). The majority of patients without bariatric surgery were male (51.2%). The most common Charlson comorbidity index score was greater than 3 in the obesity (35.13%), no obesity (31.82%), and no bariatric surgery (32.64%) groups. The most common Charlson comorbidity index score was 1 in the bariatric surgery group (27.99%). A detailed description of the baseline demographics, Charlson comorbidity index and hospital-level characteristics is provided in Table 1.

**Table 1:** Baseline hospital-level and demographics in patients with or without obesity, and with or without a history of bariatric surgery who were admitted for AF or atrial flutter. AF: atrial fibrillation

Baseline characteristics	No obesity (%)	Obesity (%)	Total (%)	p-value	No bariatric surgery (%)	Bariatric surgery (%)	Total (%)	p-value
Sex								
Male	50.62	52.54	51.03	<0.01	51.2	36.13	51.03	<0.01
Female	49.38	47.46	48.97		48.8	63.87	48.97	
Mean age								
	72.27 years	64.53 years			70.68 years	63.02 years		
Race								
White	82.22	79.98	81.74	<0.01	81.71	84.36	81.74	<0.01
Black	7.78	10.95	8.46		8.46	8.96	8.46	
Hispanic	5.8	6.07	5.85		5.87	4.28	5.85	
Asian	1.74	0.69	1.51		1.52	0.32	1.51	
Native American	0.35	0.44	0.37		0.37	0.3	0.37	
Other	2.12	1.86	2.06		2.07	1.78	2.06	
Charlson comorbidity index								
0	23.48	17.76	22.24	<0.01	22.22	24.48	22.24	<0.01
1	25.52	25.7	25.56		25.53	27.99	25.56	
2	19.19	21.23	19.63		19.61	21.01	19.63	
> 3	31.82	35.31	32.57		32.64	26.52	32.57	
Median household income								
<49999	27.02	28.97	27.44	<0.01	27.47	24.7	27.44	<0.01
50000-64999	27.06	28.18	27.3		27.28	28.88	27.3	
65000-85.999	24.67	24.8	24.7		24.67	26.83	24.7	
>86000	21.25	18.05	20.56		20.57	19.59	20.56	
Insurance type								
Medicare	73.65	57.89	70.25	<0.01	70.42	56.17	70.25	<0.01
Medicaid	5.66	9.19	6.42		6.41	7.19	6.42	
Private insurance	18.44	29.61	20.84		20.68	34.79	20.84	
Self Pay	2.26	3.32	2.49		2.49	1.85	2.49	
Hospital region								
Northeast	19.91	18.45	19.6	<0.01	19.58	21.16	19.6	<0.01
Midwest	23.13	28.19	24.22		24.17	28.72	24.22	
South	41.16	39.89	40.89		40.95	35.76	40.89	
West	15.8	13.47	15.29		15.31	14.36	15.29	
Hospital bed size								
Small	21.12	20.79	21.05	<0.01	21.05	21.03	21.05	<0.01
Medium	30.03	29.81	29.98		29.99	29.72	29.98	
Large	48.85	49.4	48.97		48.96	49.25	48.97	
Hospital location								
Rural	10.89	9.39	10.58	<0.01	10.6	8.36	10.58	<0.01
Urban	89.11	90.61	89.42		89.4	91.64	89.42	
Hospital teaching status								
No	34.64	32.35	34.16	<0.01	34.2	30.02	34.16	<0.01
Yes	65.36	67.65	65.84		65.8	69.98	65.84	

The mortality rate was significantly lower in obese patients (OR 0.646; 95% [CI 0.583-0.717; p<0.001]) when compared to the general population. However, there was no significant decrease in mortality among patients who had undergone bariatric surgery when compared to the general population (OR 0.76; 95% [CI 0.482-1.2; p=0.24]). Therefore, post-bariatric surgery patients had higher in-hospital mortality than obese patients when compared to the general population (Table 2).

**Table 2:** Adjusted mortality outcome of obesity and post-bariatric groups compared to the general population.

Mortality outcome adjusted for variables with p<0.2						
Population	Odds Ratio	Std Error	t	p-value	95% CI	
Obesity	0.646	0.342	-8.24	<0.001	0.583	0.717
Post-bariatric	0.76	0.177	-1.18	0.24	0.482	1.2

Obese patients had a longer duration of hospital stay (regression 0.219; 95% [CI 0.19-0.248, p<0.001]), required higher resource utilization (regression 3491.995; 95% [CI 2870.085-4113.905, p<0.001]), had a higher likelihood of undergoing cardioversions (OR 1.434; 95% [CI 1.404-1.465; p<0.001]), and no significant difference in the rate of endotracheal intubation (OR 1.02; 95% [CI 0.92-1.127; p=0.724]) when compared to the general population (Table 3). 

**Table 3:** Adjusted length of hospital stay, total hospital charges, endotracheal intubation, cardioversion outcomes of obesity and post-bariatric groups compared to the general population.

Length of hospital stay outcome adjusted for variables with p<0.2						
Population	Regression	Std Error	t	p-value	95% CI	
Obesity	0.219	0.15	14.67	<0.001	0.19	0.248
Post-bariatric	-0.053	0.043	-1.23	0.218	-0.137	0.031
Total hospital charges outcome adjusted for variables with p<0.2						
Population	Regression	Std Error	t	p-value	95% CI	
Obesity	3491.995	317.287	11.01	<0.001	2870.085	4113.905
Post-bariatric	577.297	840.319	0.69	0.492	-1069.801	2224.396
Endotracheal intubation outcome adjusted for variables with p<0.2						
Population	Odds Ratio	Std Error	t	p-value	95% CI	
Obesity	1.02	0.05	0.35	0.724	0.92	1.127
Post-bariatric	0.583	1.158	-2	0.046	0.343	0.99
Cardioversion outcome adjusted for variables with p<0.2						
Population	Odds Ratio	Std Error	t	p-value	95% CI	
Obesity	1.434	0.016	33.24	<0.001	1.404	1.465
Post-bariatric	1.223	0.047	5.21	<0.001	1.134	1.32

On the other hand, patients who had undergone bariatric surgery did not experience a difference in the length of their hospital stay (regression -0.053; 95% [CI -0.137-0.031; p=0.218]) and resource utilization (regression 577.297; 95% [CI -1069.801-2224.396; p=0.492]), had a lower likelihood of requiring endotracheal intubation (OR 0.583; 95% [CI 0.343-0.99; p=0.046]), but a higher likelihood of undergoing cardioversions (OR 1.223; 95% [CI 1.134-1.32; p<0.001]) when compared to the general population (Table 3).

## Discussion

The obesity group experienced a higher number of cardioversions and endotracheal intubations when compared to the general population. However, post-bariatric patients exhibited a greater in-hospital mortality rate than the obesity group when compared to the general population. This research demonstrates the persistence of the obesity paradox following bariatric surgery with respect to mortality. The full comprehension of the mechanisms behind the obesity paradox is still incomplete, and it is unclear whether this phenomenon is inherently biological or influenced by lingering confounding variables [17]. 

One biological theory to support the obesity paradox is that a higher BMI, in contrast to a healthy BMI, could potentially aid in the body's reaction to heightened catabolic stress in AF by providing a larger metabolic reserve [1]. Patients in the normal BMI range may not necessarily be in their optimal physiological state and could potentially have underlying medical issues or be experiencing proinflammatory conditions. As a result, individuals with a normal BMI may possess a decreased metabolic reserve to handle the heightened catabolic stress associated with AF [1]. Conversely, cachexia is associated with an increased risk of AF and an increased risk of poor outcomes in patients with congestive heart failure [18]. Sarcopenia, which refers to the decline in muscle mass and function, is a significant indicator of frailty, disability, and mortality among elderly individuals [19]. In congestive heart failure, sarcopenia can potentially advance to cardiac cachexia and those suffering from cardiac cachexia tend to have elevated rates of AF, potentially contributing to their increased mortality risk [19]. 

One lingering confounding variable influencing the mechanisms behind the obesity paradox could be the comorbidities of obese patients and the medications prescribed for said comorbidities. This study accounted for comorbidities by performing a multivariate analysis adjusted by the Charlson comorbidity index. However, due to a lack of access to vital clinical documents such as medications, this study holds a limited view on the understanding of how medications may impact obesity and AF outcomes. Obese individuals' increased utilization of medications targeting the cardiovascular system, including angiotensin-converting enzyme inhibitors, diuretics, statins, beta-blockers, and more recently, GLP-1 agonists and SGLT-2 inhibitors necessitate particular consideration because of their unique characteristics and varying impacts on AF. For example, oral anticoagulants are known to positively influence outcomes in obese patients with AF [20]. A study conducted by Sandhu et al. analyzed a total of 17, 913 patients for outcomes of stroke or systemic embolism, a composite endpoint (stroke, systemic embolism, myocardial infarction, or all-cause mortality), all-cause mortality, and major bleeding [20]. Cox models were utilized in their study to calculate hazard ratios (HRs) and 95% confidence intervals for different BMI and waist circumference (WC) categories while adjusting for established risk factors and treatment allocation. In their multivariable analyses, they found that higher BMI was linked to a reduced risk of all-cause mortality [overweight: HR 0.67 (95% CI 0.59-0.78); obese: HR 0.63 (95% CI 0.54-0.74), P < 0.0001] as well as the composite endpoint [overweight: HR 0.74 (95% CI 0.65-0.84); obese: HR 0.68 (95% CI 0.60-0.78), P < 0.0001] when compared to normal BMI. In the ARISTOTLE trial involving AF patients treated with oral anticoagulants, higher BMI and WC were associated with a more positive prognosis [20]. 

Another confounding variable could be that this research presupposes that individuals who have undergone bariatric surgery maintain steady weight loss and sustain it over time, leading to a reclassification from obese BMI to normal BMI. However, this assumption may not accurately represent the actual post-bariatric surgery weight changes observed in a real-world clinical environment. In 2021, a systematic literature review of 2,915 abstracts, 272 full papers, and 32 studies (25 of high and seven of fair quality) reported weight outcomes on 7,391 Roux-en-Y gastric bypass and 5,872 sleeve gastrectomy patients [21]. Their review revealed that 17.6% (95% CI 16.9-18.3), or approximately one in six patients, had a weight regain of ≥ 10% after achieving their lowest weight. Another study indicated that approximately 20 to 25% of individuals experience significant weight regain following bariatric surgery [22]. That same study found that insufficient weight loss, defined as less than 50% of the expected weight loss, emerged as the primary factor leading to eligibility for revisional bariatric surgery.

The outcomes of this research could have been impacted by undisclosed variables like fluctuations in weight over time or unaccounted confounding elements such as eating habits and adherence to dietary recommendations following bariatric surgery. A study on individuals who had gastric bypass surgery revealed a notable increase in the percentage of poor dietary choices, rising from 11% to 37% in the second year [23]. Similarly, research on patients who underwent sleeve gastrectomy showed that the rate of inadequate adherence to dietary guidelines surged to 74% by the end of the first year, mainly due to insufficient intake of fruits, vegetables, legumes, and cereals [24]. Factors contributing to patient nonadherence to healthy eating include lack of self-discipline, low motivation, limited access to nutritious food options, and the challenge of preparing healthy meals amidst busy schedules [25].

Notably, obesity paradox studies commonly define obesity by BMI; however, BMI does not account for body fat and body composition. One study found that a higher waist-to-hip ratio was associated with a higher risk of death in female but not in male heart failure patients, challenging the obesity paradox [26]. Clinical measurements such as skinfold estimates, waist circumference, and waist-to-hip ratio are data that are not available on the NIS database and are thus excluded in the analysis of this study. Other data not available on the NIS database include baseline and average BMI of patients, and average time after bariatric surgery for patients to be included in this study. Further investigation utilizing the aforementioned clinical data is warranted to confirm or deny the obesity paradox.

Therefore, despite the possible existence of the obesity paradox, its practical application is still limited. Doctors should continue to promote healthy eating habits and regular exercise among patients with AF, regardless of BMI. Physical activity and exercise programs have been associated with a reduced risk of cardiovascular issues, improved cardiometabolic factors, and weight loss by creating an energy deficit [27]. Weight loss has been shown to improve cardiovascular risk markers and reduce the likelihood of adverse cardiovascular events [28]. Achieving long-term weight loss is closely tied to a significant decrease in AF burden and the maintenance of normal heart rhythm [29]. Therefore, it is crucial to investigate the factors contributing to the obesity paradox in order to understand the role of weight loss in preventing and managing AF.

Limitations

One potential limitation of this study is the possibility of overestimating the target population size. This could be attributed to the variability in ICD code practices and the absence of readmission data. The input of ICD codes in an electronic medical record (EMR) is dependent on the user, which introduces a level of subjectivity. While it is ideal for the ICD code to be updated during each clinical visit, this may not always be the case. For instance, a patient who was previously obese but has since lost weight may still be classified as obese in the EMR if the ICD code is not updated accordingly. Consequently, this could result in an inaccurate representation of the target population size. Additionally, without access to readmission data, there is a risk of counting the same patient multiple times in the study. This is particularly relevant for obese patients with multiple comorbidities who may have several admissions. As a result, the study may inadvertently inflate the target population size by counting the same patient multiple times. 

Furthermore, this study’s data only pertains to hospitalized patients and their mortality during inpatient care. It does not encompass mortality rates within 30 days after discharge or mortality prior to admission. Therefore, this study solely captures what transpired within the hospital setting, whereas, in clinical practice, patients may pass away shortly after being discharged. The study fails to consider long-term survival rates, despite the fact that being overweight or obese during adulthood is associated with significant reductions in life expectancy and increased premature death [30]. One report showed that women aged 40 who did not smoke, experienced a decrease of 3.3 years in life expectancy due to being overweight, while men of the same age and smoking status lost 3.1 years [30]. In the same report, women aged 40 who did not smoke lost 7.1 years of life expectancy due to obesity, while men of the same age and smoking status lost 5.8 years.

Despite the aforementioned constraints, the substantial sample size of this investigation certainly boosts the statistical power of its findings.

## Conclusions

The obesity paradox is a concept that challenges the conventional understanding of the relationship between obesity and health outcomes. This investigation revealed that although the obesity group had more cardioversions and endotracheal intubations when compared to the general population, post-bariatric patients had higher in-hospital mortality than the obesity group. This research reinforces the persistence of the obesity paradox following bariatric surgery with respect to mortality. Additional prospective cohort studies are needed to explore the underlying causes of this paradox and identify the specific protective elements that may be at play in overweight or obese individuals with AF.
